# Loss of *ALK* hotspot mutations in relapsed neuroblastoma

**DOI:** 10.1002/gcc.23093

**Published:** 2022-09-13

**Authors:** Lisa M. Allinson, Aaron Potts, Angharad Goodman, Nick Bown, Matthew Bashton, Dean Thompson, Nermine O. Basta, Alem S. Gabriel, Michael McCorkindale, Antony Ng, Richard J. Q. McNally, Deborah A. Tweddle

**Affiliations:** ^1^ Wolfson Childhood Cancer Research Centre, Newcastle University Centre for Cancer, Translational & Clinical Research Institute Newcastle University Newcastle upon Tyne UK; ^2^ Newcastle Genetics Laboratory Newcastle upon Tyne Hospitals NHS Trust Newcastle upon Tyne UK; ^3^ The Hub for Biotechnology in the Built Environment, Department of Applied Sciences, Faculty of Health and Life Sciences Northumbria University Newcastle upon Tyne UK; ^4^ Department of Applied Sciences, Faculty of Health and Life Sciences Northumbria University Newcastle upon Tyne UK; ^5^ Population Health Sciences Institute Newcastle University Newcastle upon Tyne UK; ^6^ Bioinformatics Support Unit Newcastle University Newcastle upon Tyne UK; ^7^ Royal Hospital for Sick Children Bristol UK; ^8^ Great North Children's Hospital Newcastle upon Tyne UK

**Keywords:** *ALK*, loss, mutation, neuroblastoma, relapse

## Abstract

*ALK* is the most commonly mutated oncogene in neuroblastoma with increased mutation frequency reported at relapse. Here we report the loss of an *ALK* mutation in two patients at relapse and a paired neuroblastoma cell line at relapse. *ALK* detection methods including Sanger sequencing, targeted next‐generation sequencing and a new *ALK* Agena MassARRAY technique were used to detect common hotspot *ALK* variants in tumors at diagnosis and relapse from two high‐risk neuroblastoma patients. Copy number analysis including single nucleotide polymorphism array and array comparative genomic hybridization confirmed adequate tumor cell content in DNA used for mutation testing. Case 1 presented with an *ALK* F1174L mutation at diagnosis with a variant allele frequency (VAF) ranging between 23.5% and 28.5%, but the mutation was undetectable at relapse. Case 2 presented with an *ALK* R1257Q mutation at diagnosis (VAF = 39%–47.4%) which decreased to <0.01% at relapse. Segmental chromosomal aberrations were maintained between diagnosis and relapse confirming sufficient tumor cell content for mutation detection. The diagnostic SKNBE1n cell line harbors an *ALK* F1174S mutation, which was lost in the relapsed SKNBE2c cell line. To our knowledge, these are the first reported cases of loss of *ALK* mutations at relapse in neuroblastoma in the absence of ALK inhibitor therapy, reflecting intra‐tumoral spatial and temporal heterogeneity. As *ALK* inhibitors are increasingly used in the treatment of refractory/relapsed neuroblastoma, our study highlights the importance of confirming whether an *ALK* mutation detected at diagnosis is still present in clones leading to relapse.

## 1 INTRODUCTION

Neuroblastoma is the most common extra‐cranial childhood cancer accounting for 15% of all childhood cancer related deaths.^1^ Neuroblastoma arises from embryonic neural crest cells, precursors of the peripheral sympathetic nervous system. Consequently, the primary locations for neuroblastoma are the adrenal medulla and paraspinal ganglia.^2^ The median age of onset is 17 months, with around 90% of cases diagnosed before the age of 5 years.^3^ Patients are stratified into risk groups according to the International Neuroblastoma Risk Group (INRG) classification system,^4^ which includes patient age, tumor pathology, *MYCN* status, 11q status, and ploidy. The 5‐year survival rate for low‐ and intermediate‐risk patients is around 90%, but falls to 50% for high‐risk patients.^5^ Despite multimodal therapy, high‐risk patients often relapse, after which the chance of survival decreases to <10%.^5^, ^6^

In most cases, neuroblastoma results from genetic mutations in early development, with only 1%–2% being familial.^7^ Neuroblastoma is predominantly a cancer of chromosomal copy number aberrations (CNA) with typical segmental chromosome aberrations (SCAs) including losses of chromosome arms 1p, 3p, 4p, and 11q and gain of chromosome arms 1q, 2p, and 17q as reported by the SIOPEN group.^8^ *MYCN* amplification occurs in ~20% of cases and is associated with rapid disease progression and poor outcome.^4^ *ALK* amplification occurs in 2%–3% of neuroblastoma and is almost exclusively associated with co‐amplification of *MYCN*.^9^, ^10^ Somatic mutational burden increases in relapsed neuroblastoma and an increased frequency of mutations in genes involved in the RAS‐MAPK pathway, including the oncogene *ALK*, has been reported at relapse.^11^

Anaplastic lymphoma kinase (ALK) is a tyrosine kinase receptor from the insulin receptor superfamily, encoded by the *ALK* gene. *ALK* mutations are the leading cause of hereditary neuroblastoma,^10^, ^12^ but also one of the most common somatic mutations. *ALK* mutations are present in 8%–14% of all neuroblastomas at diagnosis, increasing to 26%–43% at relapse.^11^, ^13^, ^14^, ^15^ Gain‐of‐function *ALK* hotspot mutations are found in the tyrosine kinase domain and account for 85% of all *ALK* mutations, including at amino acid positions R1275, F1174 and F1245.^16^ These hotspot mutations are thought to cause ligand‐independent signaling via ALK‐activating pathways involved in cell proliferation and survival.^17^

As ALK expression is restricted in normal tissue and over‐expressed in neuroblastoma, it is an ideal target for cancer treatment. Following the success of ALK inhibitors in non‐small cell lung cancer (NSCLC), clinical trials are ongoing in patients with refractory/relapsed neuroblastoma. Promising results from phase 1/2 clinical trials have led to a phase 3 study to assess the use of crizotinib in high‐risk neuroblastoma patients at diagnosis (NCT03126916).^18^, ^19^

The role of *ALK* as a driver mutation in neuroblastoma leads to the hypothesis that loss of an *ALK* mutation at relapse is very rare. In the current study, we present two clinical cases and one paired neuroblastoma cell line where *ALK* mutations were lost at relapse while other genetic abnormalities were maintained.

## 2 METHODS

### 2.1 Patients and sample collection

The two patients in this study were enrolled on the European high‐risk neuroblastoma HR‐NBL1/SIOPEN trial (NCT01704716). This research was approved by the Children's Cancer Leukemia Group (CCLG) Tissue Bank Biological Studies Steering Group (2016 BS 03) and the Research Ethics Committee (17/NE/0025).

### 2.2 Cell lines

The paired SKNBE(1n) and the SKNBE(2c) cell lines (BE1n and BE2c) established from bone marrow aspirates from a patient at diagnosis and at relapse following treatment with cyclophosphamide, doxorubicin, vincristine and radiotherapy, were cultured in RPMI 1640 medium supplemented with 10% fetal calf serum in a 37°C, 5% CO_2_ humidified incubator as previously described.^20^

### 2.3 DNA extraction

DNA was extracted from frozen tissue samples with a tumor cell content >60% using the Qiagen EZ1 Tissue DNA kit according to the manufacturer's instructions. DNA from BE1n and BE2c cell lines, was extracted as previously described.^20^ DNA was quantified using Glomax QuantiFluor dsDNA Detection System (Promega) or the Qubit dsDNA BR kit and Qubit 3.0 Fluorometer (Invitrogen).

### 2.4 Copy number analysis

Copy number data was provided by the Newcastle Genetics Lab, Newcastle Hospitals NHS Trust. Paired neuroblastoma DNAs were analyzed using high‐resolution Illumina Infinium CytoSNP‐850k v1.1 BeadChip arrays (SNPa) (*n* = 2) or Agilent whole genome 8 × 60 K oligo array (aCGH) (ISCA version 2.0) (*n* = 2). SNP arrays were analyzed using Nexus software (Biodiscovery).

CNAs and SCAs were scored using cut off criteria described by Depuydt et al.^21^ Copy number aberrations were defined as segmental if ≥3 Mb in length and did not span the entire chromosome. Due to lower aCGH quality, Case 2 diagnosis sample was visually assessed. All copy number data was scored by at least two clinical cytogeneticists.

### 2.5 *ALK* Sanger sequencing

Detection of *ALK* mutations in the tyrosine kinase domain was completed using Sanger sequencing of exons 20–29 by the Newcastle Genetics Lab. In brief, amplicon‐based PCR was performed using standard conditions and the product was run on the ABI3500xl Genetic Analyzer (Applied Biosystems). Sequencing data was visualized by Mutation Surveyor software (SoftGenetics).

### 2.6 *ALK* Agena MassARRAY

The presence of *ALK* mutations was further assessed using a new mass‐spectrometry assay. Extracted genomic DNA samples were processed using Agena iPLEX Pro‐based methodology. Custom primers were designed using the AgenaCx Assay Design Suite v2.0 and ordered from Metabion (0.04 μM synthesis scale, desalted purification). F1174L and F1174S primers: forward ACGTTGGATGCAGACTCAGCTCAGTTAATT, reverse ACGTTGGATGTGCAGCGAACAATGTTCTGG, extension CTCTCTGCTCTGCAGCAAA. R1275Q primers: forward ACGTTGGATGTGGCCAAGATTGGAGACTTC, reverse ACGTTGGATGTGAGGCAGTCTTTACTCACC, extension TTTACTCACCTGTAGATGTCT. MassARRAY data was visualized using TyperAnalyzer4 software. Spectral data was checked for all target nucleotide positions for each sample and a no template control sample used to exclude self‐priming events.

### 2.7 Targeted next‐generation sequencing

Targeted next‐generation sequencing (T‐NGS) using a custom panel of 38 genes, including *ALK*, was performed to validate the Sanger sequencing results and identify any subclonal *ALK* mutations. Previously described by Chen et al., the panel was designed in collaboration with the SIOPEN Biology Group.^22^ The sequencing library was prepared using an Illumina TruSeq custom amplicon kit v1.5 and sequenced on an Illumina NextSeq 550. Read depth ranged from 0 to 2000 and a minimum read depth of 200 was applied.

Sequencing data quality was validated using FastQC v0.11.8 and processed using the Genome Analysis Tool Kit (GATK) best practices workflow. The sequencing reads were aligned to the b37 Human reference genome using BWA MEM v0.7.17, duplicates were marked using PICARD v2.24 and base quality scores generated by GATK v3.8.0. Mutations were called using HaplotypeCaller v3.8.0 with default parameters. Sequencing reads were visualized and the presence of strand bias checked using Integrated Genome Viewer (IGV) software (Broad Institute).

### 2.8 Whole exome sequencing

Whole exome sequencing (WES) was performed by the Genomics Core Facility (Newcastle University) on the two cell lines using the Twist Human Core Exome EF Multiplex Complete Kit according to manufacturer's instructions. Sequencing was performed on a NextSeq 550 to a read depth of ×90 with 76 bp paired‐end reads. The mean coverage was ×45 and median ×37. The coverage of the exonic regions of *ALK* was ×90 (median) and ×80 (mean). The data was processed as described for T‐NGS.

## 3 RESULTS

### 3.1 Clinical information

#### 3.1.1 Case 1

A white, male patient was diagnosed with metastatic high‐risk neuroblastoma aged 2 years and 9 months with an adrenal primary tumor, distal lymph node, bone, and bone marrow metastases. Biopsy of a supraclavicular lymph node showed stroma poor, poorly differentiated, *MYCN* non‐amplified neuroblastoma (diagnosis sample in this study). He was treated on the HRNBL‐1 trial with rapid COJEC chemotherapy (cisplatin, vincristine, etoposide, carboplatin, and cyclophosphamide), 2 cycles of topotecan, vincristine, and doxorubicin (TVD) to improve response, then surgical resection of the primary tumor, busulfan, and melphalan myeloablative therapy and autologous stem cell rescue, local radiotherapy and anti‐GD_2_ immunotherapy with 13‐cis‐retinoic acid. Unfortunately, the child relapsed 12 months after completing treatment with bone, bone marrow, and central nervous system (CNS) metastases. Bone marrow biopsy confirmed relapse (relapse sample in this study). At relapse, the patient was treated with 8 cycles of temozolomide and irinotecan (TEMIRI) with partial response at the metastatic sites, but this was followed by progression of CNS disease. The CNS recurrence was treated with a craniotomy, surgical excision, and palliative radiotherapy. Following further progression, the patient sadly died 13 months from the date of first relapse.

#### 3.1.2 Case 2

A white, male patient was diagnosed with metastatic high‐risk neuroblastoma at 1 year and 4 months of age with an adrenal primary tumor, bone, and bone marrow metastases. Biopsy of the primary tumor showed stroma poor, *MYCN* amplified neuroblastoma (diagnosis sample in this study). He was treated on the HRNBL‐1 trial and randomized to rapid COJEC chemotherapy followed by 4 cycles of TVD chemotherapy for refractory disease. This was followed by surgical resection of the primary tumor and 2 cycles of TEMIRI chemotherapy. Sadly, the patient relapsed 9 months after diagnosis and developed new CNS disease and bone metastases. He was treated at relapse with resection of a parietal lobe lesion (relapse sample in this study) and a further 8 cycles of TEMIRI and palliative craniospinal radiotherapy. Unfortunately, the child developed further progressive disease with bone metastases and died 11 months from the date of first relapse.

### 3.2 Patient *ALK* mutational screening

**FIGURE 1:**
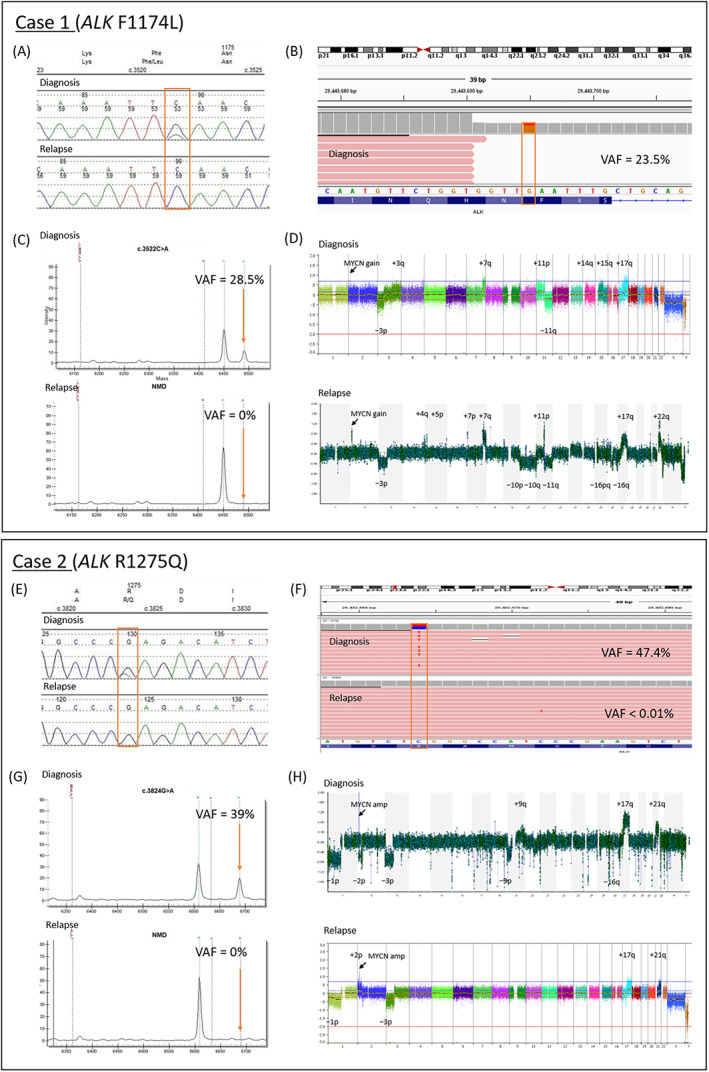
Case 1: (A) *ALK* Sanger sequencing electropherograms. (B) Targeted sequencing results. (C) *ALK* Agena MassARRAY data. (D) Copy number data, SNPa (diagnosis), and aCGH (relapse). Case 2: (E) *ALK* Sanger sequencing electropherograms. (F) Targeted sequencing results. (G) *ALK* Agena MassARRAY data. (H) Copy number data, aCGH (diagnosis), and SNPa (relapse)

*ALK* Sanger sequencing revealed an F1174L mutation in Case 1 and R1275Q mutation in Case 2 at diagnosis. Sequencing of the subsequent relapse samples showed loss of the *ALK* mutations (Figure 1). As Sanger sequencing sensitivity is limited to ~20% variant allele frequency (VAF), more sensitive detection methods were used to detect if subclonal *ALK* mutations were still present.

*ALK* Agena MassARRAY data, which has a detection sensitivity of at least 1.3% VAF, confirmed that the *ALK* F1174L mutation was present at diagnosis in Case 1 with a 28.5% VAF and undetectable at relapse. Similarly, in Case 2 the R1275Q mutation was present at 39% VAF at diagnosis but was undetectable in the relapse sample (Figure 1). T‐NGS sequencing data was available only for Case 1 diagnosis sample with 23.5% VAF. In Case 2, the R1275Q mutation VAF = 47.4%, was reduced to 0.001% (1/961 reads) at relapse (Figure 1). In further samples from Case 1, the *ALK* F1174L mutation was detectable using the MassARRAY at a VAF of 10% from a post‐chemotherapy macrodissected formalin fixed paraffin embedded sample. The *ALK* mutation was also undetectable in DNA from a further bone marrow trephine 4 months after first relapse and the CNS resection specimen, both of which had tumor cell contents of 85%–90%. DNA from the post‐chemotherapy, resected tumor sample for Case 2 also confirmed loss of the R1275Q *ALK* mutation by tNGS (G > A in 2/759 reads) with consistent SCAs.

### 3.3 Tumor copy number data

Several segmental chromosomal aberrations present at diagnosis in Case 1, including −3p, +7q, +11p, −11q, and +17q, were maintained at relapse, alongside *MYCN* gain (Figure 1), with additional SCAs gained and lost at relapse. For Case 2, the diagnosis aCGH quality was poor, however distinct SCAs were still observed. *MYCN* amplification (also detected by fluorescence in situ hybridization), and SCAs −1p, −3p, +17q, and +21q were consistent between the diagnosis and relapse samples.

Copy number data for both cases at diagnosis and relapse revealed consistent segmental aberrations, indicating that the DNA extracted from the relapsed tumors had an adequate tumor cell content.

### 3.4 Cell lines

**FIGURE 2:**
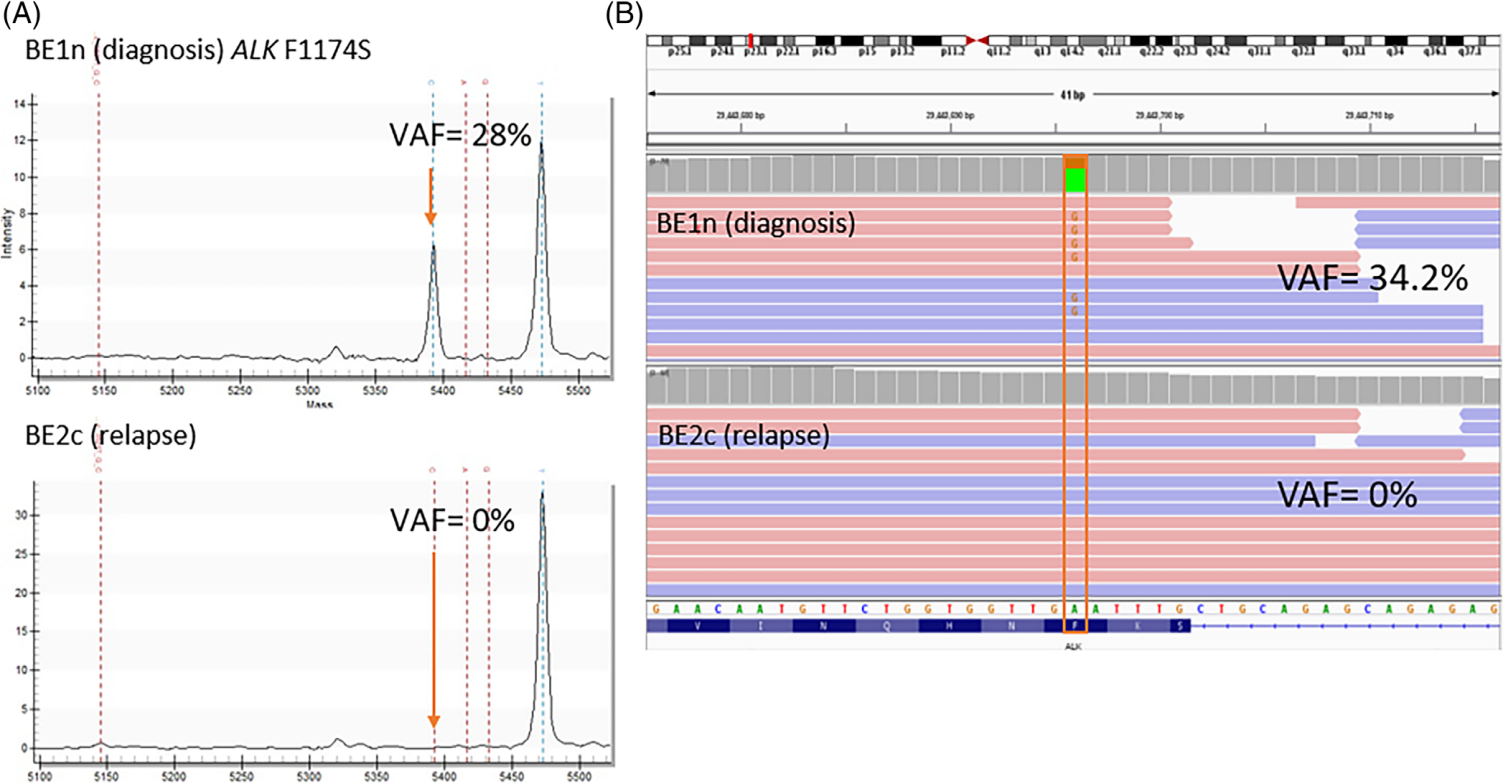
Cell lines. (A) *ALK* Agena MassARRAY data. (B) Whole exome sequencing results

WES of the paired diagnosis and relapse cell lines, BE1n and BE2c, showed loss of the *ALK* F1174S variant (VAF = 34.2%) between diagnosis and relapse (Figure 2). This was confirmed using the MassARRAY method where a F1174S VAF of 28% in the diagnostic cell line BE1n was undetectable in the relapse cell line BE2c.

## 4 DISCUSSION

In contrast to previous reports of gain of *ALK* mutations at relapse in neuroblastoma, here we present two cases where hotspot *ALK* mutations were lost at relapse. We also demonstrated this in the paired diagnosis and relapse cell lines BE1n and BE2c.

To date, the *ALK* mutation status of ~150 paired diagnosis and relapse neuroblastoma cases have been reported, alongside around 90 of our cases.^11^, ^15^, ^23^ One study showed loss of an R1274Q mutation in two out of three relapse samples from one patient, but despite histology data, there was no accompanying CNA data to confirm adequate tumor cell content in the DNA samples tested.^15^ To our knowledge, ours is the first study to report loss of *ALK* hotspot mutations at relapse in neuroblastoma with supporting CNA data.

A well‐recognized limitation of Sanger sequencing is its sensitivity, with a detection limit between 15%–20% VAF. For this reason, our initial findings using Sanger sequencing were validated using more sensitive techniques. The T‐NGS and MassARRAY methods used in this study demonstrated a limit of detection of 1.3% VAF. Using all three variant detection methods, we validated the loss of different hotspot *ALK* mutations in both patient samples and the paired cell lines.

Variation in VAF was detected between T‐NGS and Agena MassARRAY data, with the largest variation of 8.4% seen in Case 2 diagnosis, possibly due to lower DNA quality, as seen with the aCGH data. To account for T‐NGS limitations, samples were screened for quality during data processing and minimum read depth and strand bias were assessed. This was also addressed by using the MassARRAY method, which uses mass spectrometry to exploit differences in weight between variants, rather than sequencing data.

SNP array results confirmed the tumor cell content amongst samples was adequate to detect the presence of an *ALK* mutation, especially using the highly sensitive T‐NGS and MassARRAY techniques. Despite lower DNA quality in Case 2 diagnosis sample, it was possible to detect the *ALK* variant using all three detection methods and identify typical SCAs.

One neuroblastoma study reported loss of two different relapse‐specific hotspot *ALK* mutations in a patient following crizotinib treatment.^23^ Furthermore, loss of the non‐hotspot *ALK* mutation V1180L has been reported in NSCLC plasma upon treatment with lorlatinib.^24^ In these cases, *ALK* mutation loss may confer ALK inhibitor resistance. In the current study, both patients were treated on the HRNBL‐1 trial and received COJEC and TVD therapy prior to relapse, which did not involve ALK inhibitor treatment, indicating that clonal selection for wild‐type cells occurred in the absence of selection pressure from an ALK inhibitor. In addition, the BE2c cell line was established from a patient who had not received an ALK inhibitor.

The site of relapse differed from diagnosis in both cases suggesting spatial heterogeneity may have influenced the *ALK* mutation loss. However, Case 2 post‐chemotherapy sample was from the same site as diagnosis (primary tumor) and demonstrated *ALK* mutation loss. Furthermore, evidence suggests that bone marrow is more representative of the most malignant clones than other sites, as studied for Case 1 relapse.^25^ A recent study reported temporal heterogeneity of an *ALK* mutation with loss of an *ALK* R1275Q mutation in the post‐chemotherapy sample, but in this case the patient did not go on to relapse.^26^ Our study is consistent with *ALK* mutations in neuroblastoma being branch‐type rather than truncal and may not confer a survival advantage.

Interestingly both patients in the current study went on to relapse with CNS metastases. Consequently, the CNS microenvironment may have influenced clonal evolution, selecting clones without *ALK* mutations. Further research into clonal evolution of neuroblastoma in patients whose tumors lose *ALK* mutations at relapse and those with CNS relapse using clonal metastatic reconstruction and single cell analysis is required to understand this further.

## 5 CONCLUSION

Although the loss of a hotspot *ALK* mutation at relapse is rare, here we report two cases and a set of paired immortalized neuroblastoma cell lines. This novel observation due to intra‐tumoral spatial and temporal heterogeneity emphasizes the importance of confirming the continued presence an *ALK* mutation at relapse prior to the use of *ALK* inhibitors in relapsed/refractory neuroblastoma.

## CONFLICT OF INTEREST

The authors declare no conflicts of interest.

## Data Availability

The data that support the findings of this study are available from the corresponding author upon reasonable request.
